# Stereotactic Body Radiotherapy for Extracranial Oligometastatic Disease from Head and Neck Primary Cancers: A Systematic Review and Meta-Analysis

**DOI:** 10.3390/cancers16050851

**Published:** 2024-02-20

**Authors:** Adam Mutsaers, Aquila Akingbade, Alexander V. Louie, Badr Id Said, Liying Zhang, Ian Poon, Martin Smoragiewicz, Antoine Eskander, Irene Karam

**Affiliations:** 1 Department of Radiation Oncology, Odette Cancer Centre, Sunnybrook Health Sciences, University of Toronto, Toronto, ON M4N 3M5, Canada; 2 Division of Radiation Oncology, London Health Sciences, Western University, Toronto, ON M4N 3M5, Canada; 3 Department of Medical Oncology, Odette Cancer Centre, Sunnybrook Health Sciences, University of Toronto, Toronto, ON M4N 3M5, Canada; 4 Department of Otolaryngology—Head and Neck Surgery, Sunnybrook Health Sciences, University of Toronto, Toronto, ON M4N 3M5, Canada

**Keywords:** oligometastases, oligoprogression, SABR, SBRT, HNSCC

## Abstract

**Simple Summary:**

Question: Is stereotactic body radiation therapy an effective and safe treatment option for patients with oligometastatic cancer from a head and neck primary? Findings: In this systematic review and meta-analysis, stereotactic radiation demonstrated high rates of local control at 1 and 2 years (86.9% and 77.9% respectively), with no grade 4 or 5 toxicities reported. Overall survival was 80.1% and 60.7% at 1 and 2 years, respectively. Included studies were heterogeneous and of poor quality, highlighting a need for prospective studies with longer follow-up and homogeneous treatments. Meaning: Stereotactic body radiation therapy offers excellent local control and promising survival rates with acceptable toxicities for patients with oligometastatic head and neck cancers.

**Abstract:**

Introduction: Stereotactic body radiotherapy (SBRT) is increasingly used to treat disease in the oligometastatic (OM) setting due to mounting evidence demonstrating its efficacy and safety. Given the low population representation in prospective studies, we performed a systematic review and meta-analysis of outcomes of HNC patients with extracranial OM disease treated with SBRT. Methods: A systematic review was conducted with Cochrane, Medline, and Embase databases queried from inception to August 2022 for studies with extracranial OM HNC treated with stereotactic radiotherapy. Polymetastatic patients (>five lesions), mixed-primary cohorts failing to report HNC separately, lack of treatment to all lesions, nonquantitative endpoints, and other definitive treatments (surgery, conventional radiotherapy, and radioablation) were excluded. The meta-analysis examined the pooled effects of 12- and 24-month local control (LC) per lesion, progression-free survival (PFS), and overall survival (OS). Weighted random-effects were assessed using the DerSimonian and Laird method, with heterogeneity evaluated using the *I^2^* statistic and Cochran Qtest. Forest plots were generated for each endpoint. Results: Fifteen studies met the inclusion criteria (639 patients, 831 lesions), with twelve eligible for quantitative synthesis with common endpoints and sufficient reporting. Fourteen studies were retrospective, with a single prospective trial. Studies were small, with a median of 32 patients (range: 6–81) and 63 lesions (range: 6–126). The OM definition varied, with a maximum of two to five metastases, mixed synchronous and metachronous lesions, and a few studies including oligoprogressive lesions. The most common site of metastasis was the lung. Radiation was delivered in 1–10 fractions (20–70 Gy). The one-year LC (LC1), reported in 12 studies, was 86.9% (95% confidence interval [CI]: 79.3–91.9%). LC2 was 77.9% (95% CI: 66.4–86.3%), with heterogeneity across studies. PFS was reported in five studies, with a PFS1 of 43.0% (95% CI: 35.0–51.4%) and PFS2 of 23.9% (95% CI: 17.8–31.2%), with homogeneity across studies. OS was analyzed in nine studies, demonstrating an OS1 of 80.1% (95% CI: 74.2–85.0%) and OS2 of 60.7% (95% CI: 51.3–69.4%). Treatment was well tolerated with no reported grade 4 or 5 toxicities. Grade 3 toxicity rates were uniformly below 5% when reported. Conclusions: SBRT offers excellent LC and promising OS, with acceptable toxicities in OM HNC. Durable PFS remains rare, highlighting the need for effective local or systemic therapies in this population. Further investigations on concurrent and adjuvant therapies are warranted.

## 1. Introduction

Stereotactic body radiotherapy (SBRT, or stereotactic ablative radiotherapy: SABR) is increasingly used as metastasis-directed therapy (MDT) in the oligometastatic (OM) setting [1] due to mounting evidence demonstrating its oncologic benefits and safety [2], cost-effectiveness [3], and reasonable quality of life [4] associated with treatments [5]. The metachronous or de novo presentation of OM head and neck cancers (HNCs) is a rare but important entity [6], with prognosis varying by the burden of disease, histology, primary site, and patient factors [7,8].

OM HNCs are under-represented in current ‘basket-design’ prospective studies [2,9], and the relative scarcity of the presentation [7] likely precludes timely disease-specific prospective studies to inform optimal treatment decisions and prognostic discussions. While a meta-analysis of surgical metastasectomy in OM HNC patients with pulmonary metastases showed excellent 5-year overall survival (OS) [10], highlighting the potential for aggressive intervention to modify the disease course, a similar analysis for patients treated with SBRT has not yet been performed.

Given this lack of disease- and treatment-specific data, challenges exist in clinical decision making and prognostication. This systematic review and meta-analysis was performed to quantify the efficacy and safety outcomes of HNC patients with extracranial OM disease treated with SBRT. We hypothesized that the survival outcomes will be similar to the surgical series, with high rates of local control and low incidence of serious toxicity.

## 2. Methods and Materials

### 2.1. Review

A systematic review utilizing Preferred Reporting Items for Systematic Reviews and Meta-Analyses (PRISMA) guidelines [11] was conducted. Cochrane, Medline, and Embase databases were queried from inception to August 2022 for English-language studies of extracranial OM HNC patients treated with stereotactic radiotherapy. The search was executed by the lead author (A.M.), with search terms available in Appendix A. All study designs, including peer-reviewed conference abstracts, were eligible. Full inclusion criteria are displayed in Table 1. HN subsites included the nasopharynx, oropharynx, larynx, hypopharynx, a primary of unknown origin, and salivary glands. Non-English-language studies, polymetastatic patients (>5 lesions), cutaneous primaries, intracranial disease, mixed-primary cohorts failing to report HNC separately, nonquantitative endpoints, and other definitive treatments (surgery, conventional radiotherapy, and radioablation) not reporting outcomes separately were excluded. Using Covidence software (https://support.covidence.org/help/how-can-i-cite-covidence, Melbourne, Australia) for process management, independent authors (A.M. and A.A.), screened titles, abstracts, full-texts, and abstracted relevant data. Discrepancies were settled by agreement or by a third author (I.K.). The bibliographies of included studies were evaluated for relevant publications. This review was not registered in advance.

### 2.2. Appraisal

Included studies were appraised utilizing the Newcastle Ottawa Scale by two independent reviewers (A.M. and A.A.) (Supplementary Files—Table S1). Assembled studies were able to provide the retrospective evaluation of clinical outcomes, but not in a comparative fashion. Publication bias was assessed visually using funnel plots for 1- and 2-year LC and OS (Supplementary Files—Figure S3).

### 2.3. Endpoints and Analysis

Meta-analysis was performed as per Meta-analysis of Observational Studies in Epidemiology guidelines [12], with a checklist provided (Supplementary Files—Figure S4). Examined endpoints included the pooled effects of the 12-, 24-, and 36-month local control (LC) per lesion, 12-, 24-, and 36-month progression-free survival (PFS), and 12-, 24-, and 36-month overall survival (OS) and toxicity (as per Common Terminology Criteria for Adverse Events, where reported). These factors were selected due to clinical relevance and common reporting among prior studies. Weighted random-effects were assessed using the DerSimonian and Laird method, with heterogeneity evaluated using the *I*^2^ statistic (significance defined as >50%) and Cochran Qtest (significant if *p*-value < 0.10). Forest plots were generated for each endpoint. Tau-squared (τ2) represents the between-study variance.

All statistical analyses were conducted using the Meta-Analysis Package for R (version 4.2.1).

## 3. Results

### 3.1. Study Demographics

**Figure 1:**
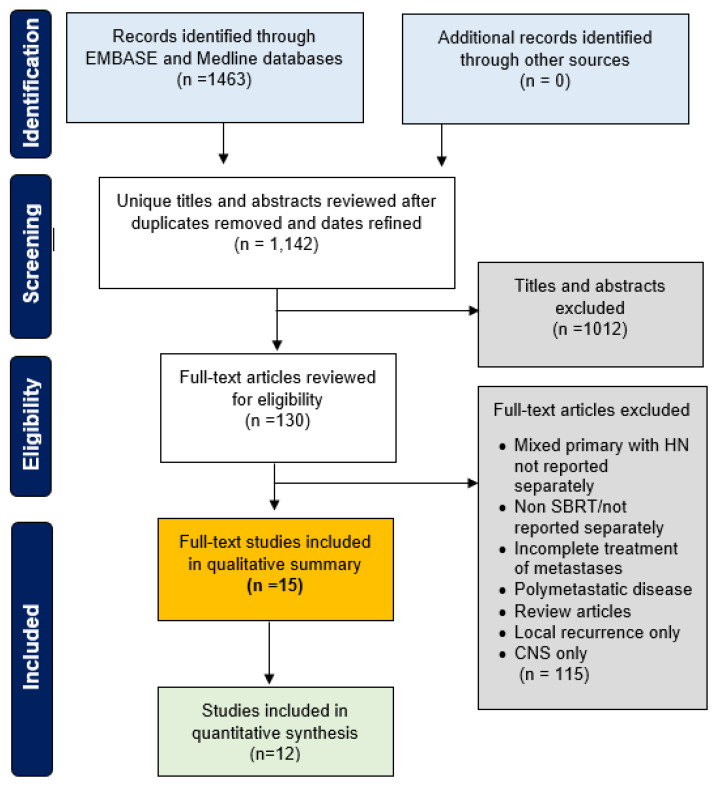
PRISMA flow diagram.

Fifteen studies met the inclusion criteria (639 patients, 831 lesions), with twelve eligible for quantitative synthesis with common endpoints and sufficient reporting. Twelve were fully published and three in abstract-only format. A PRISMA diagram is presented in Figure 1, with study demographics summarized in Table 2. Fourteen studies were retrospective, with a single prospective trial. Studies were small, with a median of 32 patients (range: 6–81) and 63 lesions (range: 6–126). The OM definition varied, with an upper limit of two to five total metastases, mixed synchronous and metachronous lesions, and a few studies that included a minority of oligoprogressive lesions (Table 2). The radiation dose, fractionation, and technique was heterogeneous, ranging from 1 to 10 fractions (20–70 Gy).

### 3.2. Study Quality

All studies were found to have significant methodologic limitations impacting the quality by the Newcastle Ottawa Scale, largely attributable to the lack of comparator cohorts in all series. Full assessment and grading can be found in Supplementary Materials. Publication bias was visually found to be modest across all evaluated endpoints, with the OS plots demonstrating more symmetry than the LC plots (Supplementary Files—Figure S3).

### 3.3. Local Control

**Figure 2:**
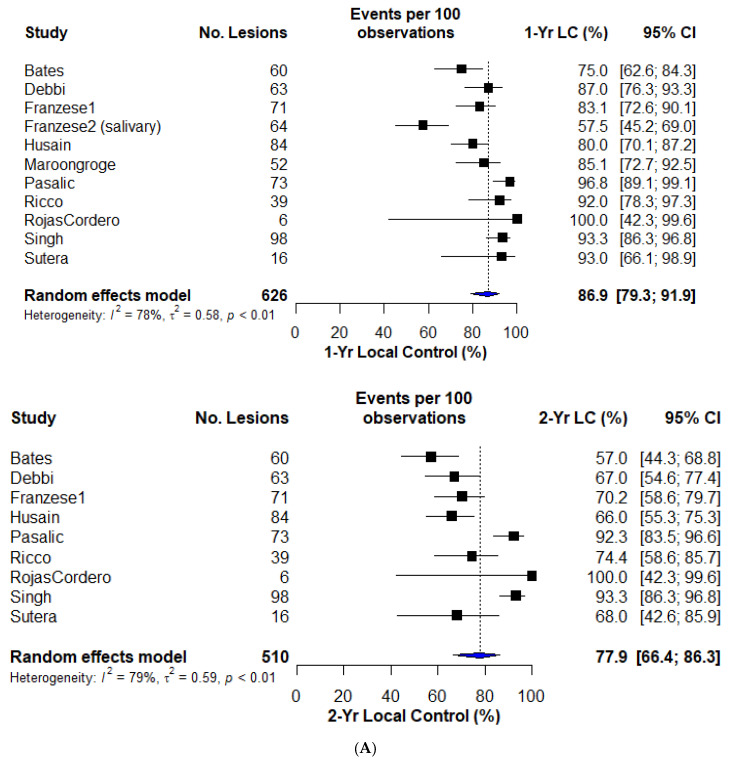

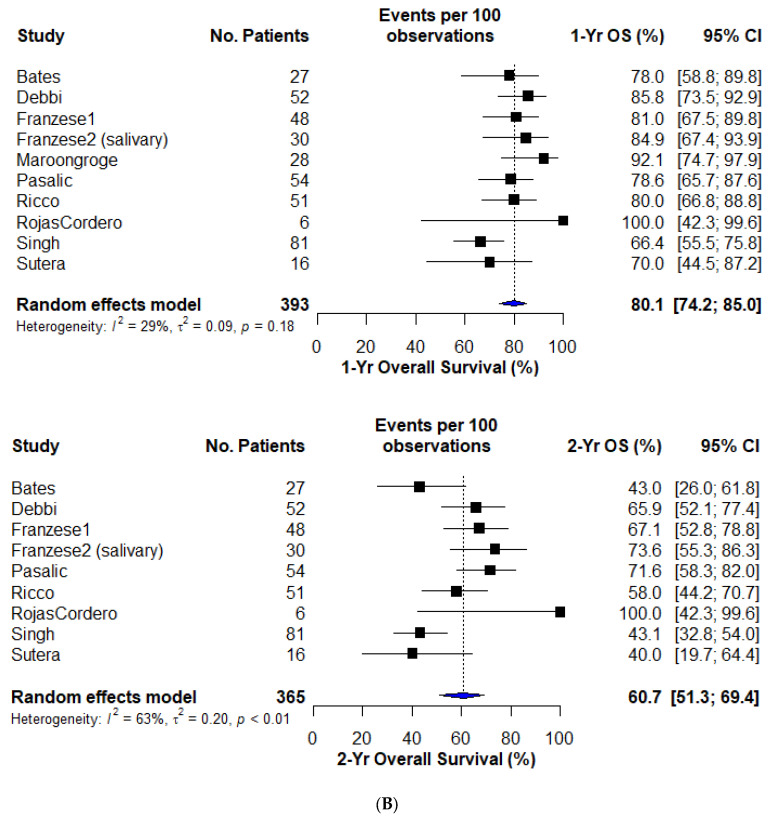
(**A**) One- and two-year local control forest plots. (**B**) One- and two-year overall survival forest plots.

The one-year LC (LC1), reported in 12 studies, was 86.9% (95% confidence interval [CI]: 79.3–91.9%). LC2 was 77.9% (95% CI: 66.4–86.3%), with significant heterogeneity across studies. Figure 2A demonstrates 1- and 2-year LC forest plots. The lowest LC was identified in a study examining only OM salivary gland malignancies (LC1: 57.5%). With few reporting studies, three year LC and OS available in Supplementary Files—Figure S2.

### 3.4. Progression-Free Survival

PFS was reported in five studies, with a PFS1 of 43.0% (95% CI: 35.0–51.4%) and a PFS2 of 23.9% (95% CI: 17.8–31.2%), and homogeneity identified across studies (Supplementary Files—Figure S3).

### 3.5. Overall Survival

OS was analyzed in nine studies, demonstrating an OS1 of 80.1% (95% CI: 74.2–85.0%) and an OS2 of 60.7% (95% CI: 51.3–69.4%). Figure 2B demonstrates 1- and 2-year OS forest plots. 

### 3.6. Toxicity

Less than half (n = 7, 47%) of the studies reported toxicity. When reported, treatment with stereotactic radiation was well tolerated with no reported grade 4 or 5 toxicities, and grade 3 toxicity rates uniformly below 5% (Table 1). The most common grade 3 toxicity was pneumonitis.

## 4. Discussion

Across heterogeneous studies, SBRT demonstrates the ability to provide excellent and durable LC with minimal toxicity. These findings are in keeping with reports of SBRT for OM in other primary disease sites, including the kidney [28], prostate [29], nonsmall-cell lung cancer [30], and breast cancer [31]. Despite limited reporting, low rates of toxicity are in keeping with other series of SBRT for OM [2]. Included studies featured predominantly metachronous presentations, OM disease in the lung, and treatment with photon-based stereotactic radiation. While these parameters are generalizable for a majority of OM HNC patients, individual studies report limited subgroup assessments. One notable outlier focusing on salivary gland primaries with a mix of radioresistant histologies [32] demonstrated a relatively poor LC and OS [18], raising the question of dose-escalation, alternate particles, and/or combination therapy for this higher-risk group.

The established standard for most metastatic HNC patients remains single-agent or combination systemic therapy, though guidelines are increasingly supportive of MDT in select, good-performance-status patients [33] with limited disease burden. Immunotherapy (alone or with chemotherapy) has demonstrated the ability to improve OS [34] while maintaining quality of life [35], though may be limited due to the modest objective response rates (30–40%), efficacy differences by programmed death ligand-1 expression, and the potential for rare but serious toxicities. Furthermore, chemotherapy options are often limited in this population due to age, performance status, and comorbidities. While no disease-specific studies have compared standard therapy with MDT, mounting randomized evidence from basket trials and other disease sites suggest oncologic advantages [9,36,37,38]. In SABR-5, a prospective single-arm study of 361 patients receiving SBRT for OM disease, only 5% were HNCs (~80% were prostate, breast, colorectal, lung, and renal) and showed impressive LC1 and LC3 rates of 93% and 87%, respectively. Further, a United Kingdom-based prospective registry study of 1422 patients who underwent SBRT for three or fewer metachronous metastases did not include any HNC patients [39]. Since prospective and randomized evidence is lacking, this systematic review and meta-analysis represents the most comprehensive analysis of HN-specific OM management. Data from the European Oncology Radiation Therapy Collaborative Oligo-Rare [40] and SABR-COMET 10 [41] trials, two actively accruing Phase III randomized studies of SBRT compared to standard care in OM patients (including HNC patients), are eagerly awaited.

The inclusion of a few oligoprogressive lesions in four studies is representative of the lack of clear direction regarding the benefit of treatment in varying cases of limited disease. The recent CURB trial randomized 106 breast or lung cancer patients with five or fewer oligoprogressive lesions after one or more lines of systemic therapy to the standard of care or SBRT to all sites of progression [42]. A substantial PFS benefit was identified in the lung cancer group (2.2 months vs. 10.0 months; *p* = 0.002), while no difference was apparent in breast cancers. While this population has more limited dedicated evidence to suggest the benefits of treatment, the practical advantages of keeping patients on well-tolerated, otherwise effective, or funded systemic therapy with limited progression are difficult to ignore in the interim. The proper classification of ‘oligo’ patients remains a challenge, with parameters varying by geography, disease site and histology, and investigator [43]. A 2020 consensus recommendation from Guckenberger et al. proposed a detailed taxonomy of standardized language to better define inclusion for trials and to more appropriately prognosticate and treat patients [44]. Future studies should attempt to distinguish the temporality, treatment status, and extent of disease to enable more clinically applicable data collection.

In patients with an excellent performance status and very limited disease, there is a debate regarding the optimal local treatment modality. While SBRT has been championed in recent years due to the demonstrated clinical success, the relative convenience, and the ability to concurrently manage multiple organ sites of disease, surgical metastasectomy has been well studied in this population (Table 3). A 2015 meta-analysis of retrospective studies which resected limited HNC pulmonary metastases (one to six, with the majority being single) showed a promising 5-year OS of 29.1% [10], with more recent series showing even better outcomes [45]. In comparison, our analysis demonstrated a 3-year OS (Supplementary Material) of 56.7% across the four studies reporting, with none reporting a 5-year OS for comparison. While the inherent selection biases limited cross-study comparison, it is important to note that surgical risks typically increase with age, comorbidities, and multiple sites of disease, while SBRT is less limited by these factors. As such, multidisciplinary discussions for individual patient treatment decisions are of paramount importance to optimizing care.

Several series reported a mixed MDT approach to OM lesions, including surgery, SBRT, radiofrequency ablation (RFA), or combinations with similarly strong outcomes (Table 3). Several of these studies compared patients who underwent MDT with those who did not, finding significant improvements in survival outcomes [48,49,51]. In the absence of randomized evidence for this population, these retrospective comparisons serve as a hypothesis generating data for future studies. Finally, while conventionally fractionated radiation remains an option for patients with OM disease, it lacks some inherent benefits of SBRT. With conventional radiation, the greater number of fractions required to deliver ‘curative-intent’ doses is less convenient for patients, potentially more costly to the health care system [3], and can delay the time to introduction or the reinitiation of systemic therapies. Further, there is interest in the potential for SBRT to induce the theoretically synergistic ‘abscopal’ effect when combined with immunotherapies, although the clinical outcomes in trials have been mixed [53,54]. Shorter palliative regimens lack the ‘ablative’ capacity of SBRT, and presumably the oncologic and local control benefits, although this has not been evaluated definitively.

The median PFS of 6.1 months is shorter than that found in some prospective studies of SBRT in OMD [9,37,55] and raises questions about the presence of a true ‘oligometastatic’ state in HNC. The retrospective nature of the included studies, the heterogeneous mix of histologies, the staging investigations, the patterns of metastases, the varied use of adjuvant systemic therapies and observation, and the varied baseline patient characteristics make it challenging to generalize our results broadly. The rates of PFS at 1 and 2 years of more than 40% and 20% (Supplementary Files—Figure S1), respectively, raise optimism that a subset may experience significant benefits from ablative therapy, with appropriate patient selection being a critical factor. Importantly, the pattern of failure at the time of progression should be examined in the future. Further oligometastatic recurrences may be amenable to further local ablative therapies, potentially inducing a longer PFS. Some have advocated for the use of a ‘second PFS’ or a ‘modified PFS’ as a better surrogate for OS in the OM population [56]. Repeat stereotactic radiation was common in the initial SABR-COMET trial, including some patients who had survival beyond five years [9], and subsequent therapy was not reported in the majority of our included studies.

Our review excluded series looking exclusively at brain metastases, a relatively rare finding in HNC primaries. Patel et al. reported on 19 patients with 38 brain metastases from HNC, demonstrating a good LC with single-fraction stereotactic radiation in a mix of intact and resected lesions (77.3% actuarial LC at 1 year) [57]. OS was limited in this cohort, with 1- and 2-year rates of 52.9% and 31.7%, respectively.

The management of the primary cancer in synchronous metastatic presentation remains a question of interest. Given the morbidity and mortality that can arise from uncontrolled primaries in the anatomically complex HN region, there is often clinical justification for radical treatment or aggressive palliation. This has been supported by a randomized trial in patients with de novo nasopharyngeal carcinoma, wherein radical treatment of the primary disease demonstrated an OS benefit of more than 20% at 2 years (*p* = 0.004) [58] at the expense of increased toxicity. Several analyses of the American Surveillance and End Result (SEER) database have shown consistent benefit to treating primary disease aggressively [59,60,61]. Similar aggressive management has shown mixed signals in other tumor sites, including potential benefits in prostate cancer [62] and no clear advantage in breast cancers [63]. Beyond the local benefits, theories have postulated the reduction in further metastatic spread [64]. Enrolled patients in trials evaluating SBRT for OM, including the SABR-COMET [2], STOMP, and ORIOLE [36] studies, were required to have treated primary tumors. The recently opened randomized Phase III SABR-SYNC trial is investigating the concurrent management of uncontrolled primary tumors with the comprehensive ablation of metastatic disease across a mix of primary sites and pathologies, and will stratify based on the extent of metastatic disease (fewer than four or greater than four lesions) and histology [65].

While there has been significant momentum in the adoption of MDT, given the evidence discussed above, there remain some reasons for caution. Not all randomized trials investigating MDT or SBRT have shown benefit. Notably, the recent preliminary reporting from the Phase II/III NRG-BR002 trial, which compared systemic therapy alone with systemic therapy with MDT, failed to show a PFS or OS benefit over 125 patients with OM breast cancers [66]. This is potentially attributable to the quality of systemic therapy alone in many patients, nullifying any benefit that MDT may have provided. In the absence of comparative data in the OM HN setting, this must also be considered. Rapidly emerging systemic therapies offer durable tumor control, while promising side-effect profiles offer excellent alternatives for some patients [67]. While serious toxicities associated with SBRT are rare, treatment is not risk-free, with grade 4 and 5 toxicities of 5% in some studies [9]. The radiation dose, planning parameters, and dose limits vary institutionally, further impacting the risk–benefit ratio from center to center. Ongoing evaluation of clinical trial options, careful patient selection and consent, and the integration of emerging data is paramount for clinicians [8].

## 5. Future Directions

The combination of SABR and immunotherapy has garnered interest in metastatic cancer due to the hypothesized ‘abscopal effect’, whereby radiation therapy triggers an immune response toward the remaining, untreated cancer cells [8]. In HNC, a randomized Phase II trial comparing nivolumab (PD1 inhibitor) with nivolumab and SBRT to a single lesion failed to demonstrate a preferential response or increase in survival [53]. Notably, patients in this trial had polymetastatic disease, and only a single site of disease was irradiated. In the OM setting, a multicenter Phase III trial investigating the combination of SBRT and camrelizumab compared to camrelizumab alone for patients with five or fewer nasopharyngeal metastasis and a controlled primary tumor is accruing data [68]. Patients in the intervention arm will receive SBRT for all sites of disease. A prospective study of 15 patients with regionally or distantly recurrent HN SCC treated with a combination of SBRT and nivolumab showed acceptable toxicity and an excellent LC at 6 months (96%) using doses of 24 Gy in three fractions [69]. Finally, an ongoing Phase I/II trial is investigating the combination of immunotherapy (durvalumab and tremelimumab) with concurrent SBRT for between 2 and 10 OM lesions, with tolerable side-effect profiles to date [70]. Another combined approach of concurrent chemotherapy and SBRT is being evaluated in patients with OM HNC in the accruing randomized Phase II study GORTEC 2014-04 trial [71]. The addition of systemic therapy to local treatment may provide more durable PFS.

Moving forward, clarifying the benefit and appropriate time to intervene in oligoprogressive patients, crystalizing the role of primary control, systemic therapy following SBRT, and the evaluation of dose escalation or heavy-particle therapy for radioresistant histologies will be of interest.

## 6. Limitations

This review is limited by the included study characteristics. Authors were only able to interpret English-language studies. Prospective data were limited and included studies were of low methodologic quality. As such, strong rates of LC, reasonable OS, and low toxicity rates could be partially attributable to selection bias. Furthermore, limitations in reporting in both included and excluded studies prevented subgroup reporting or the analysis of histology or SBRT site-specific outcomes, which may harbor important prognostic differences. Human papilloma virus-associated pathology was not detailed in most studies, negating the opportunity to examine the potential differences in the failure patterns and prognosis in this subgroup that have been raised in other studies [6,72]. While our study is limited by the retrospective nature of most included articles, site- and histology-specific randomized data may be challenging to accrue for HNC. In the absence of quality prospective evidence, this analysis may provide useful data to inform clinical decision making, prognostication, and future research questions.

## 7. Conclusions

This pooled analysis of heterogeneous OM HNC studies demonstrates that SBRT offers strong LC and promising OS, with acceptable toxicities in OM HNC, building on surgical evidence that aggressive MDT may be warranted selectively in this population. Durable PFS remains rare, highlighting the need for effective systemic or sequential local therapies in this population. Further investigation on concurrent and adjuvant therapies, dose escalation in radioresistant histologies, combinations of other MDTs, and prospective, comparative series to confirm the efficacy and safety are needed to compliment the role of radiation in these patients. Subgroup analyses from cohort studies and from basket-histology randomized trials may also be of interest in defining the ideal role for SBRT in HNC patients.

## Figures and Tables

**Table 1:** PICOS.

Population	Patients with metastatic (synchronous or metachronous) cancers from head and neck primaries (mucosal or parotid) with less than or equal to 5 total lesions to any anatomic site in the body (excluding the brain)
Intervention	Stereotactic radiation therapy, defined as highly conformal, image-guided, high dose-per-fraction (>=6 Gy/fraction, total BED >= 48 Gy) external beam radiation therapy delivered with ablative intent
Control	Multiple-arm studies in which one or more arms involved stereotactic radiation or no control group
Outcomes	Primary outcome: local control at 1 and 2 years. Secondary outcomes: overall survival at 1 and 2 years; progression-free survival at 1 and 2 years; any toxicity
Study Design	Prospective or retrospective studies with greater than 5 head and neck cancer patients

**Table 2:** Included study demographics.

Study	Pts/ Lesions	Design	OM HNC Definition	Sites Treated	Age (Med)	Performance Status	RT Dose	BED10 (Min–Max)	Median Follow-Up	LC		OS		Toxicity
										12M	24M	12M	24M	
Bates [13]	27/60	Retro	1–5 mets, mixed histology; (22 metachronous, 5 synchronous; OP:5)	Mixed sites; 59% lung only	65 (20–76)	-	35/5# to 50/5#	59.5–100 Gy	19.2 mos	75.0%	57.0%	78.0%	43.0%	NR
Bonomo [14]	27/28	Retro	1–5 mets, HNSCC; <5cm max dim, (de novo:22; OP:6)	Lung only	67 (37–85)	ECOG 0–2	26Gy/1# to 54Gy/3#	93.6–151.2	22.0 mos	NR	NR	NR	NR median OS of 47mos	14.8% Gr1–2
Debbi [15]	52/63	Multi-institutional/Retro	1–2 mets, HNSCC; all metachronous, <5 cm max dim	Lung only	65.5 (50–83)	ECOG 0–2	60Gy/3#	180 Gy	45.3 mos	87.0%	67.0%	85.8%	65.9%	2% Gr2 2% Gr3
Dohopolski [16]	17/NR	Retro	1–5 mets, mixed histology, majority metachronous	Lung only	68 IQR (69–75)	-	Mixed: 60/3# to 48/4#	105.6–180 Gy	29.5 mos	HR		HR		-
Franzese [17]	48/71	Retro	1–5 mets, mixed histology; metachronous: 42; OP: 6	Mixed sites; 59% lung	70.5 (32–83)	ECOG 0–1	21Gy/3# to 75Gy/8#	35.7–145.3 Gy	20.2 mos	83.1%	70.2%	81.0%	67.1%	1.7% Gr2
Franzese [18]	30/64	Retro	1–3 mets, salivary gland primary	Mixed sites; 53% lung	56.5 (25–82)	-	20/1# to 54/5#	60–115.5 Gy	29.2 mos	57.5%		84.9%	73.6%	NR
Hong [19]	34/NR	Multi-institutional/Retro	1–5 mets, NS	NR	62.7 IQR: (54–71) *	-	24Gy/3# to 50Gy/10#	43.2–75.0 Gy	26.2 mos	HR		HR		NR
Husain [20] (ABS)	42/84	Multi-institutional/Retro	<=5 extracranial mets; HNC mixed histology; metachronous: 31; synchronous: 11	Mixed site; 50% lung	64 (NR)	-	20–28/1# to 50 Gy/10 (median BED = 100)	60–100 Gy (median BED = 100)	18.2 mos	80%	66%			4.7% Gr 3 pneumontiis
Maroongroge [21] (ABS)	28/52	Retro	Limited spine mets, mixed histology;	Spine only	–	-	-	-	51.7 mos	85.1%		92.1%		NR
Pasalic [22]	54/73	Retro	1–3 mets, mixed histology; majority metachronous (nonoligo patients also reported)	Lung only	65 (26–93)	-	Range from 50/4# to 70/10#	Range from 112.50 to 119.0 Gy	20 mos	96.8%	92.3%	78.6%	71.6%	6.2% Gr2
Ricco [23]	51/39 **	Multi-institutional database/Retro	1–3 mets, mixed histology	Lung only	69 (18–93)	KPS 90 (25–100)	Median: 50 Gy/3#	Median: 50 Gy/3#	13.0 mos	92.0%	74.4%	80.0%	58.0%	NR
Rojas Cordero [24] (ABS)	6/6	Retro	1–4, mixed histology; <5 cm max dim	Lung only	75 (24–94)	-	50 Gy/5# to 55/5#	100.0–1115.5 Gy	42.0 mos	100%	100%	100%	100%	NR
Singh [25]	81/98	Registry/Retro	HNC OMD–NS	Mixed site; 53% lung	68 (NR)	KPS 90	20 Gy/1# to 60 Gy/5	Median BED: 37.5–180 Gy med: 92.2 Gy	NR	93.3%	93.3%	66.4%	43.1%	17.3% Gr 1–2, no Gr3+ no Gr 3
Sutera [26]	16/16	Phase II –single-arm	1–5 mets, mixed histology;	Mixed site	66.4 IQR: (59.5–74.6)	KPS 90 (60–100)	41–54 Gy in 3–5 #	97.0–104.0 Gy	41.3 mos	93.0%	68.0%	70.0%	40.0%	7.5% Gr2 2.0% Gr3
Yamamoto [27]	NR/126	Multi-institutional database/Retro	1–5 mets, NR	Lung only	72 (63–78) *	ECOG 0–3	NR	BED > 75 Gy		HR		HR		NR

NR = not reported; HR = outcomes for sub-population reported only as Hazard Ratio; NS = not specified/no further demographic data available; # = number of fractions of radiation; Retro= retrospective cohort study; SBRT = stereotactic body radiation therapy; OM = oligometastasis; OS = overall survival; * = represents data from overall cohort, not specific to HNC population; ** = 51 patients with HNC in mixed cohort, LC data reported on 39 HNC lesions.

**Table 3:** MDT beyond SBRT.

Series	Design	Metastasis-Directed Therapy Utilized	Number Patients/ Lesions	Demographics	Key Findings
Vincent [45]	Retrospective review	Surgical metastasectomy	81/81	Single distant metastasis	5-year OS: 40%
Young [10]	Systematic review and meta-analysis of retrospective studies	Surgical metastasectomy of lung metastases	11 studies; 387/NR	Lung-only oligometastasis, 1–6 nodules resected per patient (286 with single)	5-year OS: 29.1%
Beckham [46]	Retrospective single-institution	MDT included surgery, RT, RFA—most (74) received no treatment	104 */248 (30 underwent MDT)	Mixed cohort of OM and PM, with mixed treatment	5-year OS in patients receiving MDT = 31%
Weissman [47]	Retrospective single-institution	90% SBRT, 25% surgery, 3% RFA	40/75	1–7 mets, lung in 58%; 68% metachronous	LC1 = 90%, LC3 = 85% (no difference between modality)
Shulz [48]	Retrospective review with propensity-matched cohort	SBRT and or surgery (radiation dose/fractionation not detailed)	37/64	Limited metastatic disease from HNSCC	Significantly higher OS (23.97 months vs. 7.07 months) for patients receiving MDT
Lardinois [49]	Retrospective single-institution	Surgery (26), radiation (dose/technique not specified) (10), chemotherapy (47), supportive care (17)	100/123	Majority lung metastases, <5, 94% metachronous	RFS–OS and OS were significantly better than patients without specific treatment (respectively, *p* = 0.02 and *p* = 0.002)
Li [50]	Retrospective single-institution; Propensity-matched	Chemo + RFA	37/66	Nasopharyngeal carcinoma with <=3 liver metastases; 22 metachronous; 15 synchronous	Median OS 32.5 months vs. 18.8 months (chemo-only matched cohort). 29.5% 5-year OS in chemo + RFA group
Wright [51]	Retrospective single-institution	Metastatic patients presenting after surgery treated with surgery or RT 14 additional OM patients treated with systemic therapy	12/16	<5 metastasis, with most having 1 or 2	Significantly better OS in patients treated with MDT than systemic therapy alone (not reached vs. 40.7 months)
Poonia [52]	Retrospective single-institution	Skeletal muscle metastases treated with surgery/RT/chemo	6/6 *	Mixed cohort of OM and PM, with mixed treatment	Limited sample of rare entity limits findings

NR = not reported; MDT = metastasis-directed therapy; SBRT = stereotactic body radiation therapy; RFA = radiofrequency ablation; OM = oligometastasis; OS = overall survival; PM = polymetastatic disease. * Patients with OM disease (<5 distant mets).

## Supplementary Materials

The following supporting information can be downloaded at: https://www.mdpi.com/article/10.3390/cancers16050851/s1, Table S1: Study appraisal—Newcastle Ottawa Scale; Figure S1: One- and two-year progression-free survival; Figure S2: Three-year local control and overall survival; Figure S3: Risk of Publication Bias for Local Control and Overall Survival Endpoints; Figure S4. Meta-analyses of Observational Studies Checklist.

## Appendix A

### Appendix A.1. Search Strategy

#### Appendix A.1.1. PubMed

((((((((((((((Stereotactic body radiation therapy) OR (Stereotactic body radiotherapy)) OR (SBRT)) OR (Stereotactic Ablative Radiation)) OR (Stereotactic) OR (SABR)) OR (Metastasis directed therapy)) AND Head and Neck neoplasms)) OR (Head and Neck Cancer)) OR (HNSCC)) OR (Oropharyn*)) OR (Nasopharyn*)) OR (hypopharyn*)) OR (laryn*)) AND (Oligometasta*)) OR (Oligo metasta*)) OR (Oligo-metasta*)) OR (Oligoprog*))

Filters: English, Human

Results: 847

#### Appendix A.1.2. EMBASE

(head and neck neoplasms.mp. or head and neck cancer.mp. or exp head and neck squamous cell carcinoma or HNSCC.mp. or exp larynx cancer or exp hypopharynx cancer/ or exp larynx cancer/ or exp nasopharynx cancer or exp oropharynx cancer and (radiosurgery.mp. or exp radiosurgery/ or exp stereotactic radiosurgery/ or exp stereotactic body radiation therapy/ or stereotactic.mp. or sbrt.mp. or sabr.mp.) and exp metastasis or oliogmetastatic.mp or oliogmetastasis.mp or oliogmetas*.mp or oligoprogress*.mp

Limits: English language, Human

Results: 616

There were no results in the Cochrane Library.
