# Supplementary material for: Stereotactic Body Radiotherapy for Extracranial Oligometastatic Disease from Head and Neck Primary Cancers: A Systematic Review and Meta-Analysis
Source: Cancers (Basel). 2024 Feb 20;16(5):851. doi: 10.3390/cancers16050851 (PMC10930866; doi:10.3390/cancers16050851)
Supplement: Supplementary file 1 [file cancers-16-00851-s001.zip › cancers-2722396-supplementary.pdf]

cancers MDPI

Supplementary Materials: Stereotactic Body Radiotherapy for Extracranial Oligometastatic Disease from Head and Neck Primary Cancers: A Systematic Review and Meta-Analysis

Adam Mutsaers, Aquila Akingbade, Alexander V. Louie, Badr Id Said, Liying Zhang, Ian Poon, Martin Smoragiewicz, Antoine Eskander and Irene Karam

Table S1. Study Appraisal- Newcastle Ottawa Scale.

Study	Selection				Comparator	Outcome			Total Score	Quality Assessment
	Representative of Exposed Cohort	Selection of External Control	Ascertainment of Exposure	Outcome of Interest Absent at Start	External Control	Outcome	Sufficient Folllow-Up	Follow-Up Accounted		
Bates	**1**	0	1	1	0	1	0	1	5/8	**Poor**
Bonomo	0	0	1	1	0	1	0	1	4/8	**Poor**
Debbi	0	0	1	1	0	1	1	1	5/8	**Poor**
Doha	0	0	1	1	0	1	1	1	5/8	**Poor**
Franzese1	1	0	1	1	0	1	1	1	6/8	**Poor**
Franzese2	1	0	1	1	0	1	0	1	5/8	**Poor**
Hong	0	0	1	1	0	1	1	1	5/8	**Poor**
Husain	1	0	1	1	0	1	0	1	5/8	**Poor**
Maroongroge	0	0	1	1	0	1	1	1	5/8	**Poor**
Pasalic	1	0	1	1	0	1	0	1	5/8	**Poor**
Ricco	0	0	1	1	0	1	0	1	4/8	**Poor**
Rojas Cordero	0	0	1	1	0	1	1	1	5/8	**Poor**
Singh	0	0	1	1	0	1	0	0	3/8	**Poor**
Sutera	1	0	1	1	0	1	1	1	6/8	**Poor**
Yamamoto	0	0	1	1	0	1	1	1	5/8	**Poor**

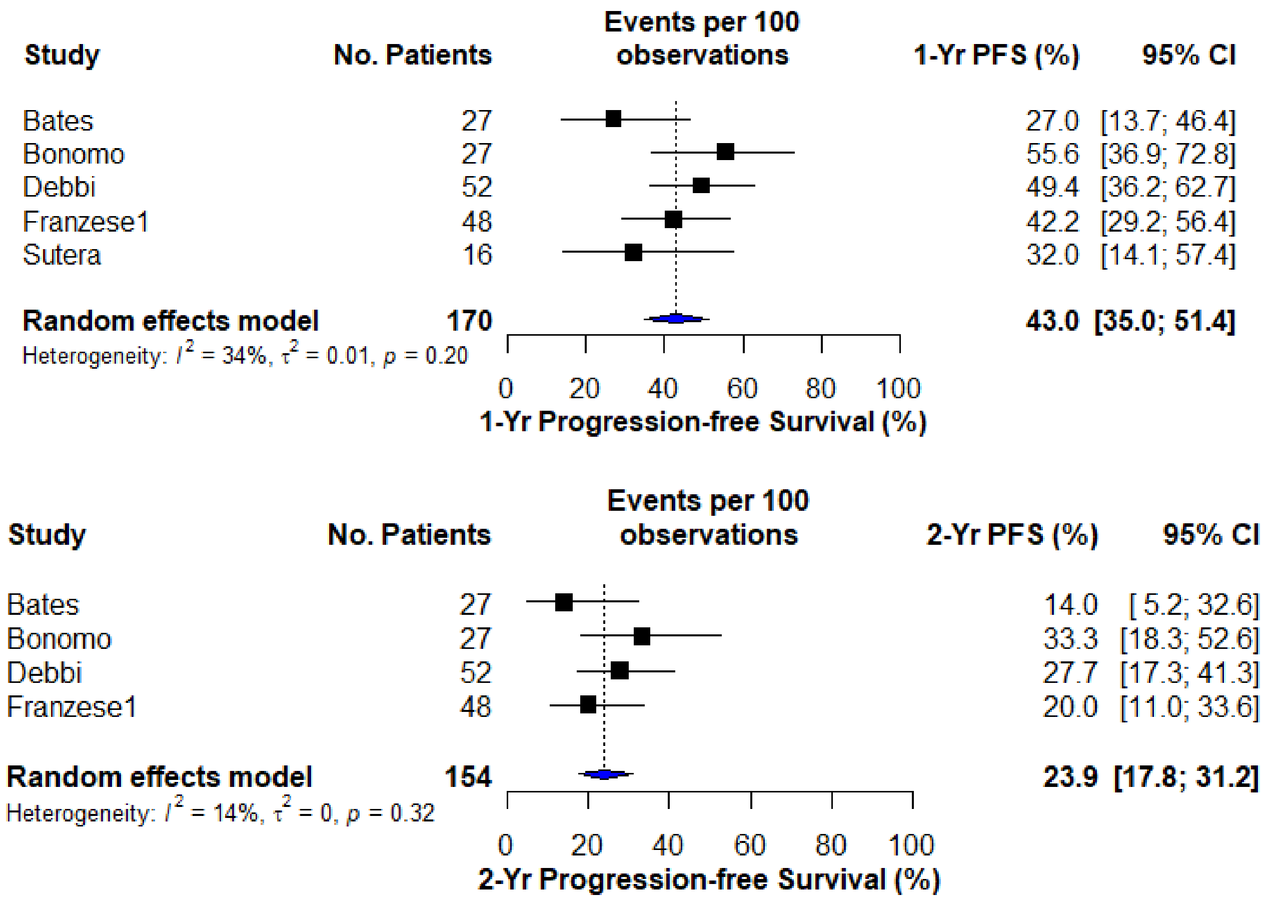

Figure S1. 1- and 2-year Progression Free Survival.

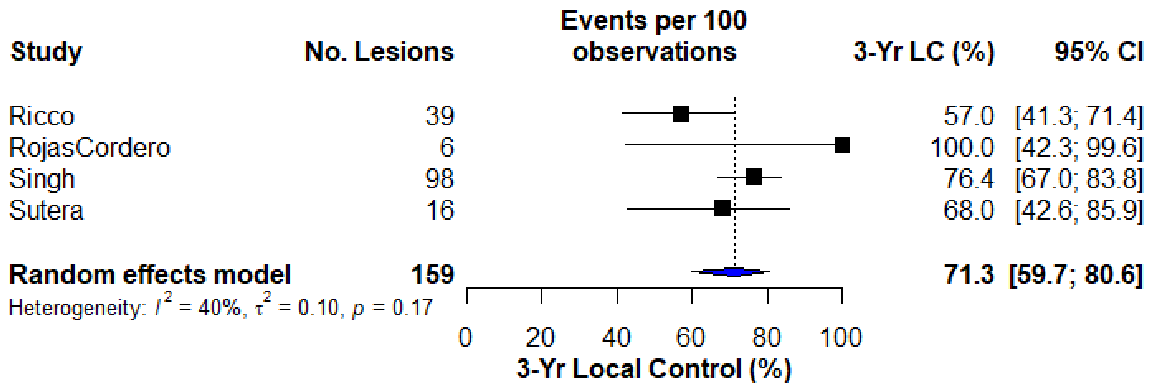

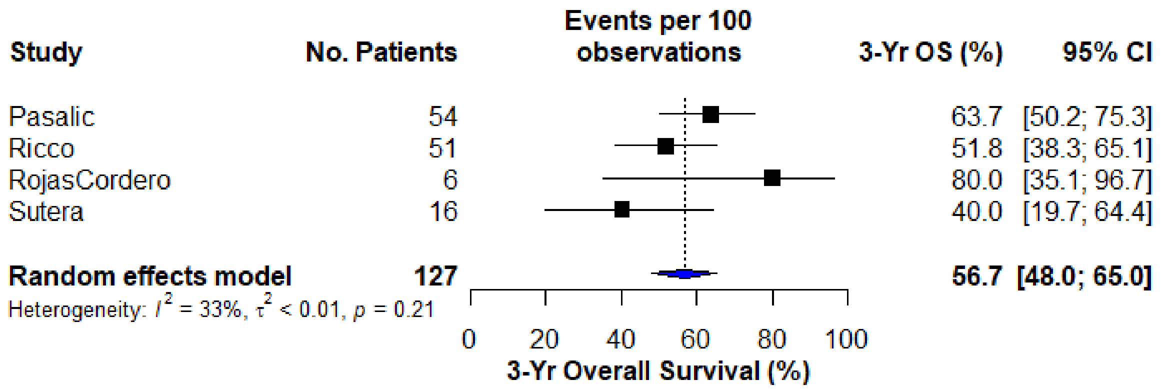

Figure S2. 3-Year Local Control and Overall Survival.

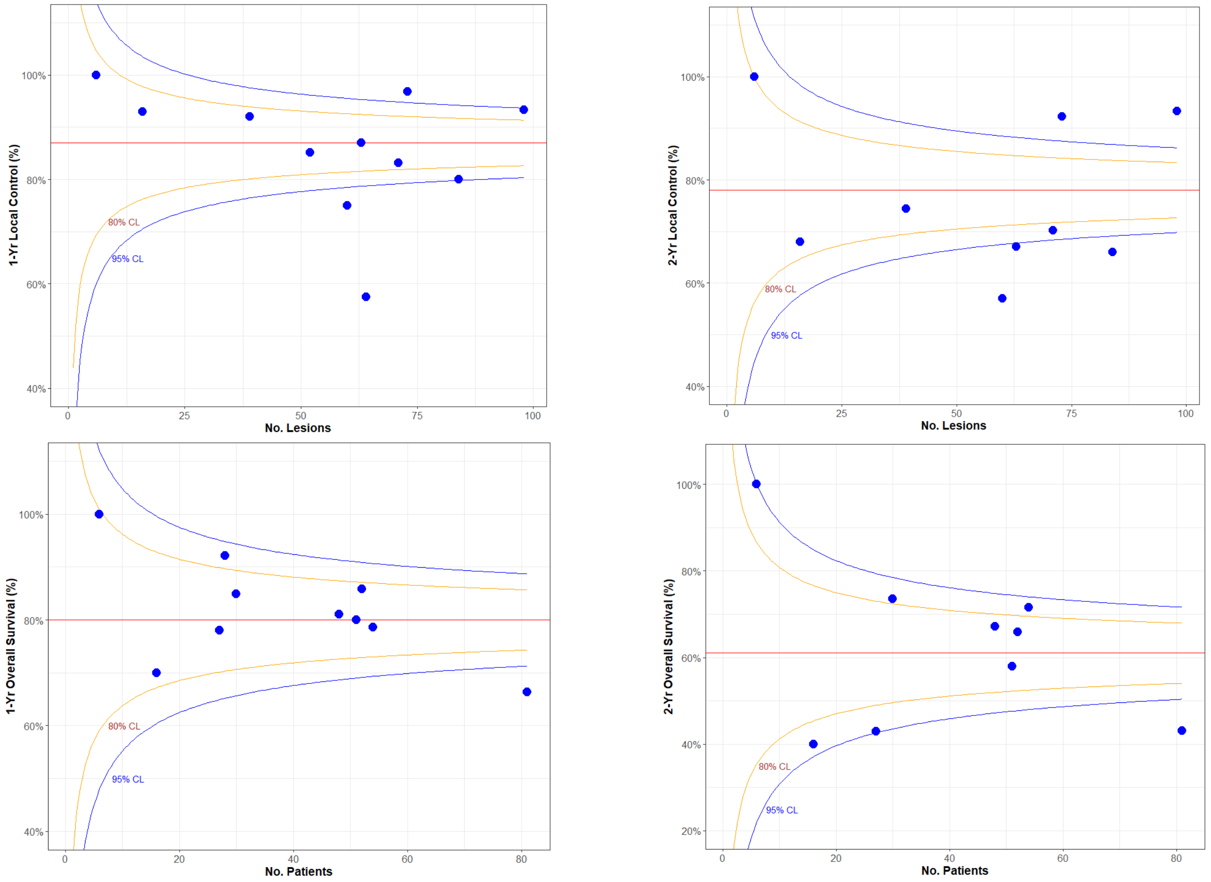

Figure S3. Risk of Publication Bias for Local Control and Overall Survival Endpoints. Funnel plots were generated by plotting on the the 1-Yr or 2-Yr local control or overall survival on the y-axis, and number of patients from each study on the x-axis. The endpoint (%) from all studies is displayed as benchmark (red solid line) in the funnel plot, 80% and 95% confidence limits (CL) are also displayed (orange and blue solid lines). Each dot indicates 1-Yr or 2-Yr Overall Survival from an individual study.

Figure S4. Meta-analyses of Observational Studies Checklist

MOOSE (Meta-analyses Of Observational Studies in Epidemiology) Checklist

A reporting checklist for Authors, Editors, and Reviewers of Meta-analyses of Observational Studies. You must report the page number in your manuscript where you consider each of the items listed in this checklist. If you have not included this information, either revise your manuscript accordingly before submitting or note N/A.

Reporting Criteria	Reported (Yes/No)	Reported on Page No.
**Reporting of Background**		
Problem definition	Yes	Page 4
Hypothesis statement	Yes	Page 4
Description of Study Outcome(s)	Yes	Page 5
Type of exposure or intervention used	Yes	Page 4+5
Type of study design used	Yes	Page 4
Study population	Yes	Page 4
**Reporting of Search Strategy**		
Qualifications of searchers (eg, librarians and investigators)	Yes	Page 4
Search strategy, including time period included in the synthesis and keywords	Yes	Page 4/App A
Effort to include all available studies, including contact with authors	Yes	Page 4
Databases and registries searched	Yes	Page4
Search software used, name and version, including special features used (eg, explosion)	Yes	Page 4
Use of hand searching (eg, reference lists of obtained articles)	Yes	Page 5
List of citations located and those excluded, including justification	Yes	Table 3
Method for addressing articles published in languages other than English	Yes	Page 4
Method of handling abstracts and unpublished studies	Yes	Page 4
Description of any contact with authors	Yes	Page 4
**Reporting of Methods**		
Description of relevance or appropriateness of studies assembled for assessing the hypothesis to be tested	Yes	Page 5
Rationale for the selection and coding of data (eg, sound clinical principles or convenience)	Yes	Page 5
Documentation of how data were classified and coded (eg, multiple raters, blinding, and interrater reliability)	Yes	Page 4
Assessment of confounding (eg, comparability of cases and controls in studies where appropriate	Yes	N/A-no control

Reporting Criteria	Reported (Yes/No)	Reported on Page No.
Assessment of study quality, including blinding of quality assessors; stratification or regression on possible predictors of study results	Yes	Page 5
Assessment of heterogeneity	Yes	Page 5
Description of statistical methods (eg, complete description of fixed or random effects models, justification of whether the chosen models account for predictors of study results, dose-response models, or cumulative meta-analysis) in sufficient detail to be replicated	Yes	Page 5
Provision of appropriate tables and graphics	Yes	Yes- fig1/tab1
**Reporting of Results**		
Table giving descriptive information for each study included	Yes	Table2
Results of sensitivity testing (eg, subgroup analysis)	Yes	Page 5
Indication of statistical uncertainty of findings	Yes	Page 5
**Reporting of Discussion**		
Quantitative assessment of bias (eg, publication bias)	Yes	Page 5
Justification for exclusion (eg, exclusion of non–English-language citations)	Yes	Page 10
Assessment of quality of included studies	Yes	Page 6
**Reporting of Conclusions**		
Consideration of alternative explanations for observed results	Yes	Page 7
Generalization of the conclusions (ie, appropriate for the data presented and within the domain of the literature review)	Yes	Page 10
Guidelines for future research	Yes	Page 9
Disclosure of funding source	Yes	Title page

Once you have completed this checklist, please save a copy and upload it as part of your submission. DO NOT include this checklist as part of the main manuscript document. It must be uploaded as a separate file.
